# A Method for Locating Growth Points of Cucurbitaceae Plug Seedlings Based on Structured Light Vision

**DOI:** 10.3390/jimaging12080342

**Published:** 2026-07-28

**Authors:** Yang Zheng, Wu Chen, Zihao Xu, Qingcang Yu

**Affiliations:** School of Computer Science and Technology, Zhejiang Sci-Tech University, Hangzhou 310018, China; 2023220603108@mails.zstu.edu.cn (Y.Z.); wcworkzstu@163.com (W.C.); 202230603127@mails.zstu.edu.cn (Z.X.)

**Keywords:** 3D reconstruction, point cloud processing, plant phenotyping, target localization, light plane calibration, agricultural robotics

## Abstract

To address the difficulty in growth point localization caused by cotyledon overlapping and leaf occlusion during plug seedling grafting, a localization method based on grid structured light is proposed. An acquisition system consisting of a complementary metal-oxide-semiconductor (CMOS) camera and a grid structured light laser projector is established. A multi-depth plane calibration method is adopted to fit the light plane equation for each laser line. To address the grid line discontinuity problem, a coding method based on three-dimensional constraints of light planes is proposed. The cotyledon point cloud is reconstructed by combining the light plane equations with the camera model, and a circumscribed triangle is constructed to approximate the arc center of the fan-shaped point cloud for growth point localization. Experimental results show that the average interlayer error of light plane calibration is 0.059 mm. For 50 non-overlapping single seedlings, the average localization error is 1.68 mm with a success rate of 100%. For 150 overlapping seedlings, the success rate reaches 89%, outperforming the traditional ellipse fitting method (68%). The proposed method can provide reliable growth point localization information for grafting robots.

## 1. Introduction

Plug seedling grafting is a critical task in facility agriculture. Accurate localization of the growth point is key to achieving automated grafting. Traditional grafting supply processes largely rely on manual labor, which is not only intensive but also inefficient, making it difficult to meet the demands of industrialized seedling production [1,2].

In recent years, machine vision has been widely adopted for characterizing grafted seedlings. The methods can be broadly categorized into two groups: visible-light image-based detection and active vision-based 3D sensing. The former includes elliptical Hough transform for cotyledon contour fitting [3,4], ordered contour chain methods [5], and parameter extraction methods [6]. These approaches perform well on single, non-occluded seedlings. However, they rely on contour segmentation from 2D images, which becomes unreliable under cotyledon overlap, leaf occlusion, or uneven illumination, leading to a significant increase in failure rate. Binocular vision obtains depth information through stereo matching, but its accuracy degrades in scenes with sparse or repetitive leaf textures.

The latter category mainly includes line-structured light and RGB-D cameras. Line-structured light can provide high-precision profile measurements but typically requires a scanning mechanism, making it difficult to perform one-shot localization in a static top-down view. RGB-D cameras, such as Kinect and RealSense, can simultaneously output color images and depth maps [7]. However, these sensors rely on active infrared projection and integrated depth decoding, which are prone to pixel saturation on specular surfaces such as plant leaves, leading to depth information failure. These methods each have limitations when applied to the specific task of growth point localization for plug seedlings. Therefore, there is a need for a method that can acquire 3D information of cotyledon surfaces in a single shot from a top-down perspective, to overcome the challenges posed by occlusion and cotyledon overlap.

Structured light technology actively projects coded patterns and can acquire 3D information even in low-texture or occluded scenes. Yu et al. [8] reviewed vision-based optical 3D reconstruction techniques and their applications in crop sensing. Peng et al. [9] developed a handheld structured light scanner for maize and successfully obtained point clouds and leaf area measurements in the field. Wu et al. [10] applied line-structured light to 3D reconstruction and parameter extraction of plug trays. However, as mentioned above, line-structured light requires a scanning mechanism and is not suitable for one-shot localization in a static top-down view.

Unlike line-structured light, grid-structured light projects a two-dimensional grid pattern and can capture denser 3D information in a single frame, making it suitable for fast measurement in static scenes [11]. According to the coding strategy, grid-structured light can be divided into coded and non-coded types. Coded grid-structured light embeds specific codewords (e.g., color coding, width coding) into the projected pattern to enable unique identification of individual light stripes [12]. However, this typically requires large-volume DLP projectors or digital light processing devices, which are difficult to integrate into compact systems. In contrast, non-coded grid-structured light uses a fixed-pattern laser pointer as the projection source, which is compact and low-cost, but the identity of each light stripe in the projected pattern is ambiguous and requires post-processing for coding and matching. The grid-structured light laser projector (21 × 21 grid) used in this work belongs to this non-coded category.

Non-coded grid-structured light faces three major challenges when applied to plant surface measurement. First, laser image incompleteness. The cotyledons of plug seedlings are typically higher than the tray surface, and the soil and tray walls strongly absorb laser light, making the laser lines projected on background regions difficult to capture. As a result, only partial laser lines on the cotyledon surfaces are present in the captured images, with severe overall grid loss that prevents the direct establishment of a complete grid topology. Second, surface curvature-induced line spacing variation. Due to the curved morphology of cotyledon surfaces, the actual distances between adjacent laser lines change with surface undulations after projection. Traditional coding methods based on fixed distance thresholds are effective in planar scenes but are not robust on curved surfaces, often leading to line indexing errors. Third, vein reflection-induced false laser lines. The white veins on cotyledon surfaces appear as bright stripes under red laser illumination, resembling real laser lines. These false lines are difficult to distinguish from real laser lines in images and, if not handled properly, can severely interfere with subsequent line extraction and coding. Despite these challenges, previous studies have demonstrated the feasibility of grid-structured light for 3D reconstruction, including single-shot reconstruction based on light plane constraints [13] and binocular measurement using non-coded grid patterns [14,15]. However, these methods were not specifically designed for the curved and occluded surfaces of plant cotyledons, motivating the dedicated line extraction and coding strategies proposed in this work.

These three challenges make it difficult to directly apply traditional non-coded grid-structured light methods to 3D reconstruction of cotyledon surfaces, necessitating dedicated line extraction and coding strategies. To address these issues, this paper adopts grid-structured light and proposes a growth point localization method for plug seedlings. The main contributions are as follows: (1) a grid-structured light acquisition system is constructed and a multi-depth-plane-based light plane calibration method is proposed; (2) to address incomplete grid lines and uneven line spacing on cotyledon surfaces, a coding method based on three-dimensional constraints of light planes is developed; (3) based on the fan-shaped structure of the cotyledon point cloud, a triangle-approximation method for growth point localization is designed.

## 2. Materials and Methods

### 2.1. Image Acquisition System

To achieve top-down three-dimensional detection of grafted plug seedlings, a grid-structured light-based image acquisition system was constructed. The system mainly consists of an image acquisition unit, a grid projection unit, and a software development environment, with the following specific configurations.

The image acquisition unit used was a RER-USBGS1200P01 USB camera (Rervision, Shenzhen, China) equipped with a CMOS sensor. The image resolution was set to 960 × 540 pixels, and the camera was fitted with a 4.3 mm fixed-focus lens. The grid projection unit was a 21 × 21 grid-structured light laser projector (Leilan Laser, Wuhan, China) with a wavelength of 650 nm.

**Figure 1:**
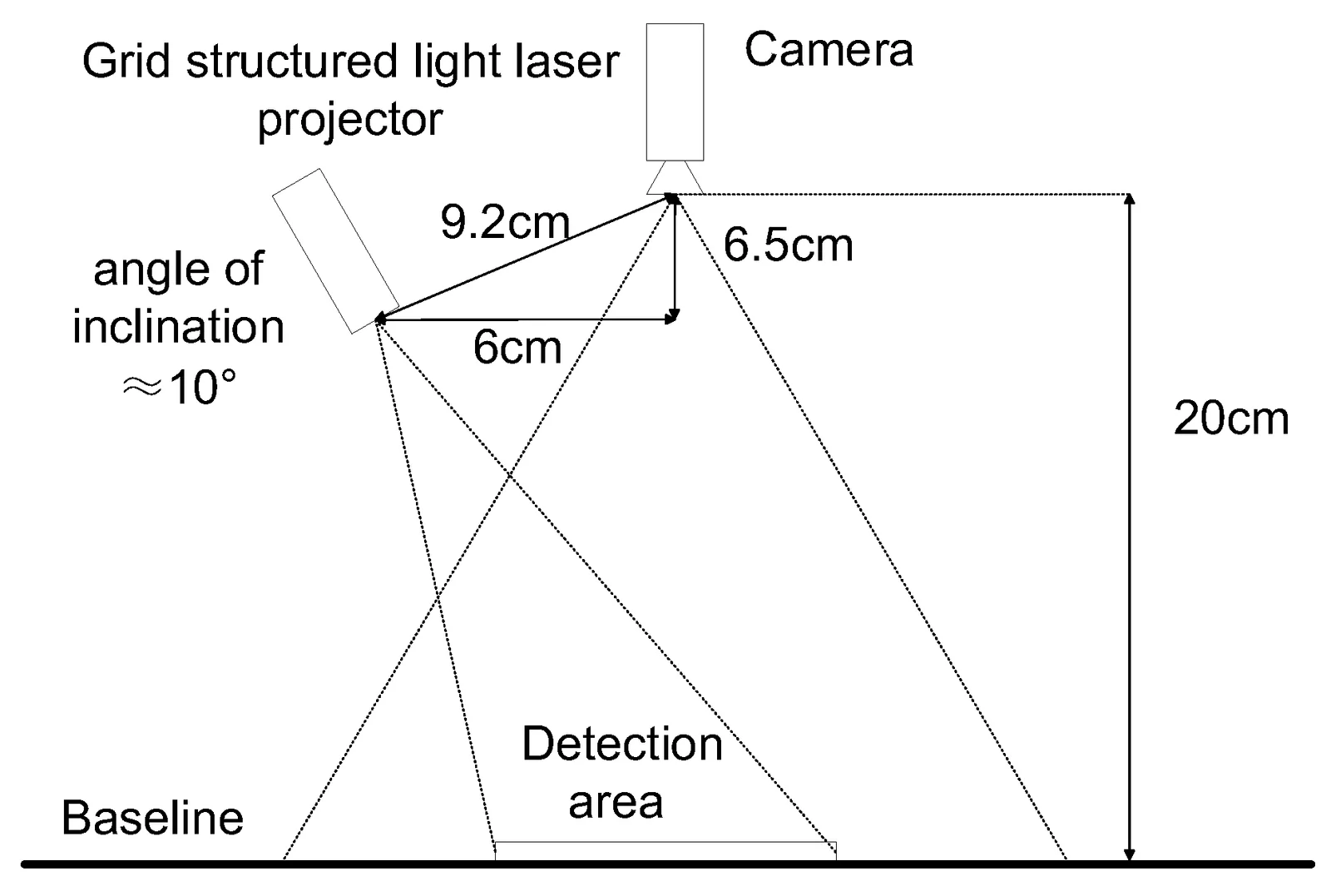
Schematic diagram of the experimental setup.

In terms of the setup, the camera was fixed 20 cm directly above the plug tray, with the lens pointing vertically downward toward the tray surface. The laser pointer was placed on the right side of the camera, with horizontal offsets of 6 cm and 2 cm in the two lateral directions, and a vertical offset of 6.5 cm below the camera, resulting in a baseline distance of approximately 92 mm between the camera optical center and the laser outlet. The projection optical axis was inclined at about 10° relative to the vertical direction. A schematic diagram of the image acquisition setup is shown in Figure 1.

The entire machine vision system was implemented in C++ using Microsoft Visual Studio 2022. Internal image processing algorithms and 3D data storage and processing relied on the Open Source Computer Vision Library (OpenCV) 4.5.5 [16]. Subsequent core operations, such as point cloud segmentation, growth point localization, and growth direction recognition, were performed using the Point Cloud Library (PCL) 1.15.1 [17].

### 2.2. System Calibration

System calibration is a fundamental prerequisite for converting pixel coordinates to 3D spatial coordinates and ensuring the accuracy of subsequent point cloud generation. It mainly consists of camera calibration and light plane calibration, providing precise parameter support for growth point detection and overlapping seedling segmentation.

#### 2.2.1. Camera Calibration

The primary goal of camera calibration is to establish the mapping relationship between image pixel coordinates and 3D camera coordinates, as well as to eliminate the influence of lens distortion on measurement accuracy, thereby obtaining the camera intrinsic matrix and distortion coefficients. The well-known Zhang’s calibration method was adopted [18], which is simple to operate, offers high calibration accuracy, and is suitable for machine vision inspection scenarios. A black-and-white checkerboard was used for calibration, with a square size of 3 mm × 3 mm and 9 × 12 inner corner points. The checkerboard was placed flat on the experimental platform, and 20 images were captured from different positions and angles to ensure that the calibration board covered various regions of the camera’s field of view.

The calibration functions provided by OpenCV were applied to the 20 captured images to estimate the camera intrinsic matrix and distortion coefficients.

The camera intrinsic matrix K is defined as

(1) $$K=\left(\begin{array}{ccc} f_{x} & 0 & c_{x}\\ 0 & f_{y} & c_{y}\\ 0 & 0 & 0 \end{array}\right)$$

where $f_{x}$ and $f_{y}$ are the focal lengths in the x and y directions (in pixels), and $\left(c_{x},c_{y}\right)$ is the principal point coordinate (in pixels).

This intrinsic matrix was used for subsequent point cloud generation and growth point localization. The estimated intrinsic parameters and distortion coefficients are listed in Table 1, where $k_{1}$, $k_{2}$ are the radial distortion coefficients, and $p_{1}$, $p_{2}$ are the tangential distortion coefficients. The average reprojection error of the calibration was 0.198 pixels, indicating that the calibration accuracy meets the requirements for subsequent 3D reconstruction.

#### 2.2.2. Light Plane Calibration Based on Multi-Depth Planes

**Figure 2:**
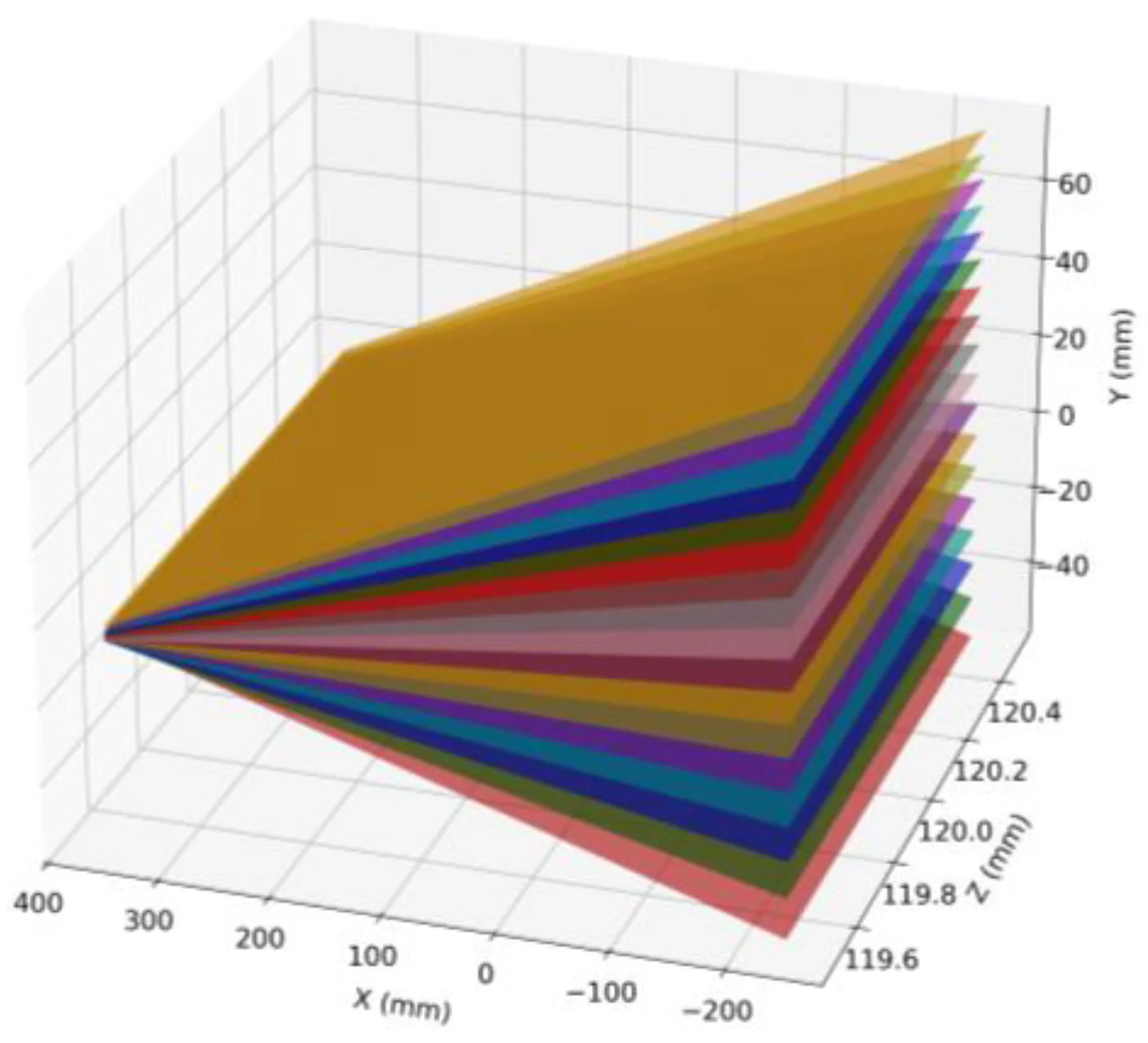
Calibration results of the horizontal light planes.

Unlike programmable projectors, a fixed-grid-structured light laser projector cannot directly provide projection matrix parameters. Therefore, a multi-depth plane calibration method was adopted: each laser line was independently fitted with its corresponding light plane equation in the camera coordinate system. The checkerboard was placed at different depths (Z = 118–122 mm), and the exact depth of each plane was obtained from the camera extrinsic parameters. For each plane, the grid laser image was processed by extracting the red (R) channel, applying Gaussian filtering, adaptive threshold binarization, and removing the overexposed central region. The skeleton was extracted using the Zhang–Suen thinning algorithm [19], and horizontal and vertical lines were separated using 8-neighborhood region growing. The central bright spot was used as a reference for numbering the laser lines (horizontal lines are numbered upward/downward as −1, −2, …, +1, +2, …; similarly for vertical lines). The pixel points of each laser line were back-projected to 3D space using the camera intrinsic parameters, yielding 3D point sets on different depth planes. Finally, the light plane equation for each line was obtained via least-squares fitting, and the consistency of the light planes was verified. The calibration results for the horizontal light planes are shown in Figure 2. In this visualization, the *X*-axis tick interval is 50 mm, the *Y*-axis interval is 20 mm, and the *Z*-axis (depth) interval is 0.2 mm. Unequal aspect ratios are used for the three axes to clearly distinguish the layered light planes; therefore, no global scale bar is provided, and the actual physical size can be accurately read from the axis ticks.

## 3. 3D Point Cloud Reconstruction Using Grid-Structured Light

### 3.1. Laser Line Extraction from Reference Images

**Figure 3:**
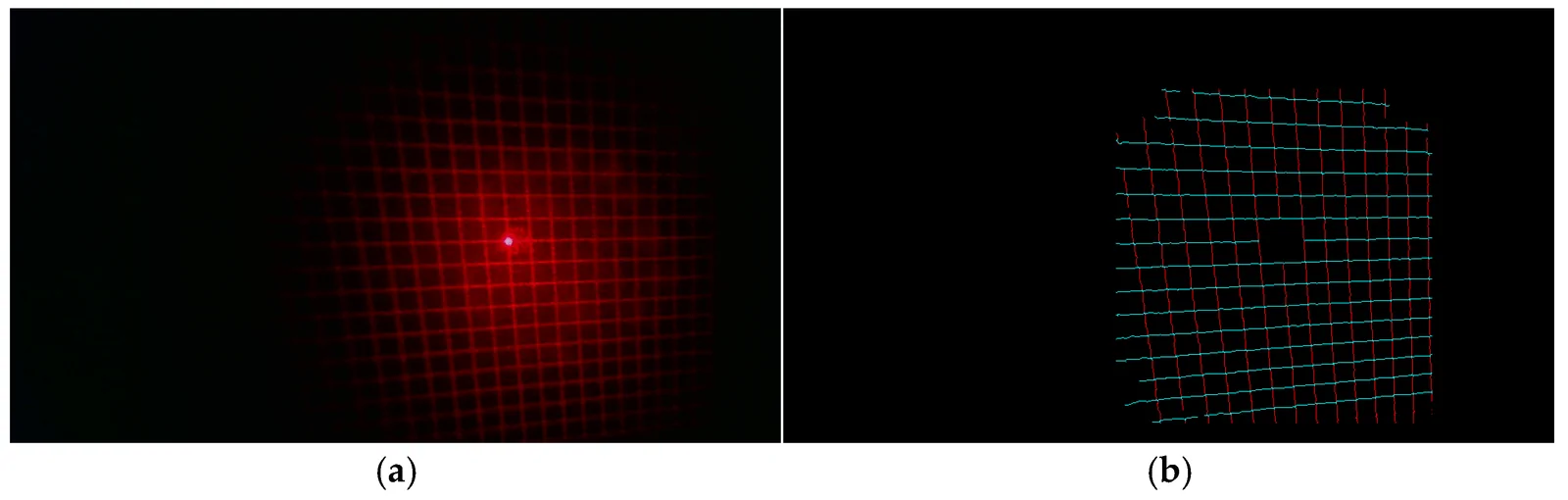
Processing results of the reference image. (**a**) Original grid image; (**b**) extracted line results.

Reference images are used for light plane calibration. Due to the limited projection angle, the brightness of some vertical lines on the left side of the image is relatively low (see Figure 3a), but the central region remains complete and clear, which is sufficient for calibration purposes. In this study, six vertical laser lines on the left side, seven vertical lines on the right side, and several horizontal lines near the center were selected for processing. The extraction pipeline (R-channel extraction, Gaussian filtering, adaptive thresholding, morphological operations, thinning, and line separation) is the same as described in Section 2.2.2 and is not repeated here. The processing results are shown in Figure 3. In Figure 3b, the blue and red lines represent the extracted horizontal and vertical laser lines, respectively.

### 3.2. Laser Line Extraction from Cotyledon Regions

Extracting laser lines from cotyledon surfaces is challenging due to vein interference and leaf curvature. A dual-image acquisition strategy was therefore adopted: images with and without laser projection were captured simultaneously, and the cotyledon mask obtained from the image without laser projection was used to constrain the extraction region.

(1) Cotyledon region segmentation: For the image without laser projection, the Excess Green (ExG) index was used to enhance contrast [20,21], followed by Otsu’s thresholding and morphological closing to obtain a binary cotyledon mask [22].
(2) Laser line extraction and false line removal: For the image with laser projection, the same pipeline as described in Section 2.1 was applied to extract the laser skeleton. The cotyledon mask was then morphologically eroded (using a 5 × 5 structuring element, twice, based on tests of 3 × 3, 5 × 5, and 7 × 7 kernels with 1 to 3 iterations on sample images; the 5 × 5 kernel with 2 iterations provided the best balance between removing boundary artifacts and preserving valid laser lines).

**Figure 4:**
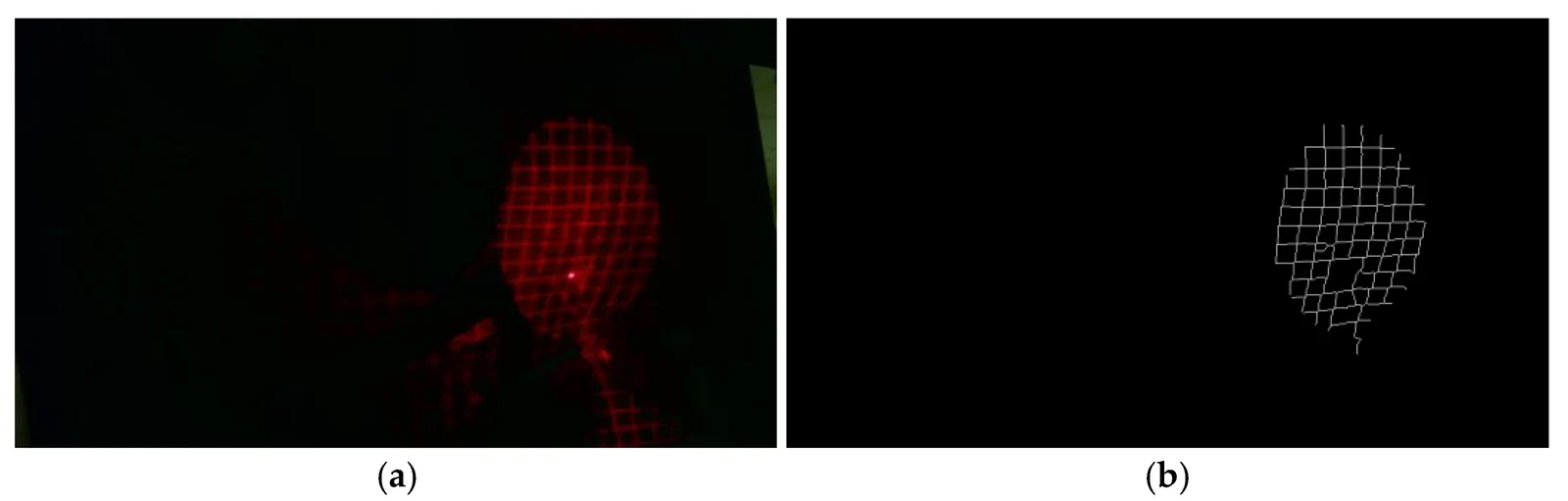
Processing results of cotyledon images. (**a**) Original image with laser projection; (**b**) valid laser lines after skeleton extraction and mask-based cropping.

Figure 4 shows the processing results for cotyledon images, where (a) is the original image with laser projection, and (b) is the final set of valid laser lines after skeleton extraction (following the pipeline in Section 2.1) and mask-based cropping.

### 3.3. Laser Line Coding Method Based on 3D Constraints of Light Planes

In point cloud reconstruction based on triangulation, a key step is to determine the light plane index corresponding to each extracted laser line. Due to the curvature of cotyledon surfaces, vein interference, and inter-leaf occlusion, the projected grid lines often exhibit uneven spacing, breaks, distortion, and incompleteness. Traditional coding methods that rely on fixed step sizes or regular patterns are not robust enough for this scenario.

Building on the basic coding strategy described in Section 2.2.2, this paper proposes a coding method based on three-dimensional constraints of the light planes. The core idea is as follows: the intersection of two perpendicular laser lines corresponds to a unique 3D point in the camera view. Using the respective light planes of the two lines, the 3D coordinates of this intersection pixel can be computed. The two resulting 3D points should satisfy a spatial consistency criterion (Euclidean distance below a preset threshold), i.e., they are considered the same point. This allows the laser lines to be accurately matched with their corresponding light planes.

**Figure 5:**
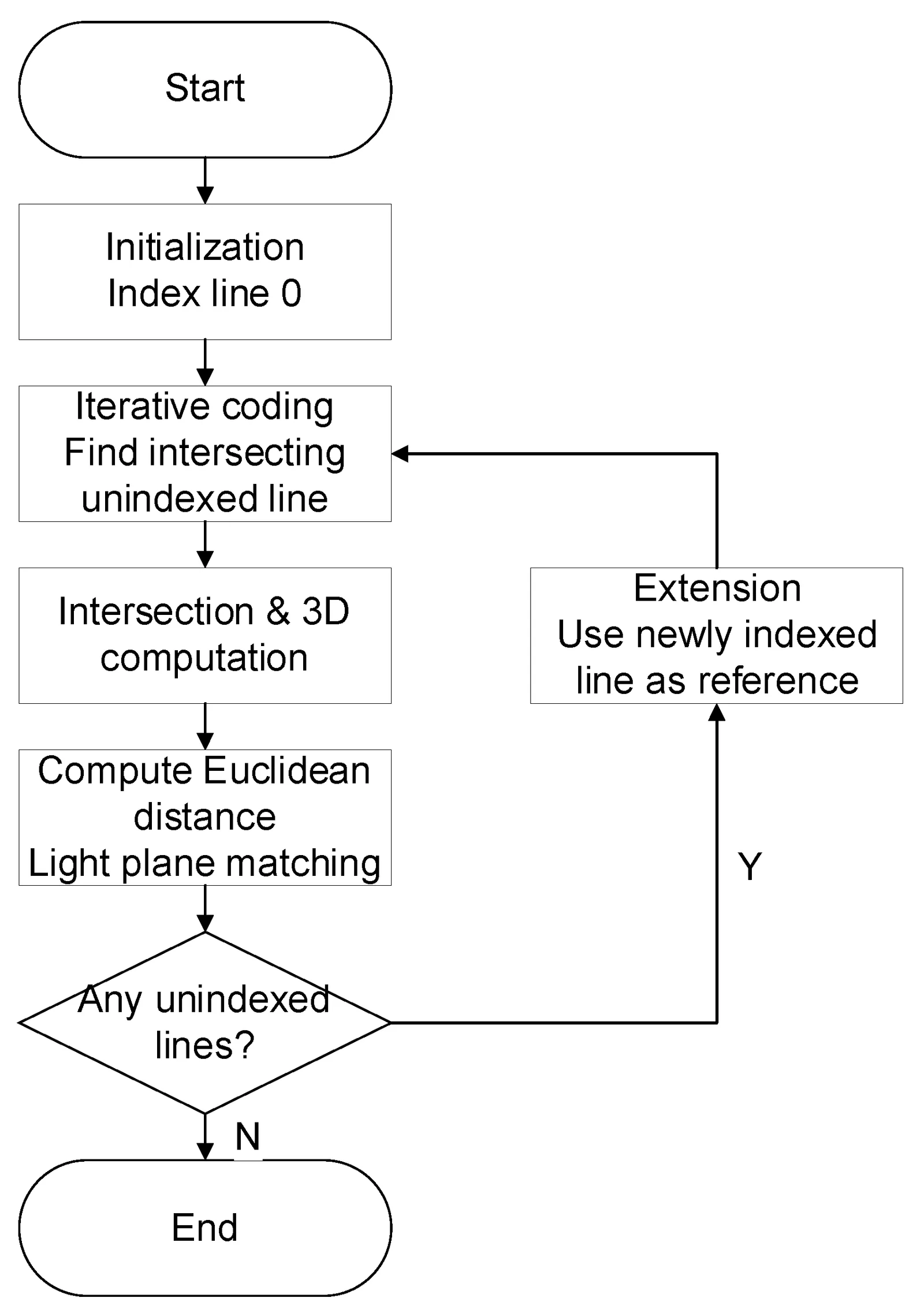
Flowchart of the coding algorithm based on 3D constraints of light planes.

The coding algorithm is illustrated in Figure 5, and the specific steps are as follows:

(1) Initialization: Locate the centroid coordinates of the central bright spot (laser center). Because the overexposed central region causes breaks in the horizontal and vertical lines of index 0, these lines cannot be directly extracted as complete segments via connected component analysis. Therefore, from the preprocessed horizontal and vertical lines, those that pass through the neighborhood of the central spot (a circular region of radius R centered at the spot) are selected. Within this neighborhood, the distance between each candidate line and the central spot is computed, and the horizontal and vertical lines with the smallest distances are chosen as the index-0 lines. The radius R was experimentally set to 25 pixels, based on tests of R = 20, 25, and 30 pixels on sample images; R = 25 pixels gave the most stable zero-line identification.
(2) Iterative coding: Using an already coded line (e.g., a horizontal line) as a reference, find an intersecting vertical line that has not yet been coded.
(3) Intersection localization and 3D computation: Locate the pixel of the intersection between the two lines and compute its 3D coordinates using the light plane of the known horizontal line.
(4) Light plane matching: Traverse all calibrated vertical light planes. For each candidate vertical light plane, compute the 3D coordinates of the same intersection pixel and calculate the Euclidean distance between this computed point and the previously obtained 3D point. The vertical light plane that yields the smallest distance (below a threshold τ mm, determined experimentally by testing values of 1, 2, 3, and 4 mm on sample images; τ = 2 mm gave the highest correct matching rate and was adopted) is selected as the correct match, and the vertical line is successfully coded.
(5) Extension: Add the newly coded line as a new reference and repeat steps (2)–(4) until all valid laser lines have been coded.

This method does not rely on line continuity or fixed spacing, effectively handles vein interference, line breaks, and height variations, and provides a robust coding scheme for incomplete grid laser lines. The resulting line-to-light-plane correspondences serve as a solid foundation for subsequent point cloud reconstruction.

### 3.4. Cotyledon Point Cloud Reconstruction via Triangulation

After coding, for each pixel of a laser line, the corresponding light plane equation and the camera back-projection model are combined to solve for the 3D coordinates of that point. By iterating over all valid laser pixels, the point cloud of the cotyledon surface is generated using Equations (2)–(4):

(2) Ax+By+Cz+D=0

(3) $$\lambda \left[\begin{array}{c} u\\ v\\ 1 \end{array}\right]=K\left[\begin{array}{c} x\\ y\\ z \end{array}\right]$$

(4) $$\left\{\begin{array}{c} z=\frac{-D}{A\cdot \frac{u-u_{0}}{f_{x}}+B\cdot \frac{v-v_{0}}{f_{y}}+C}\\ x=\frac{(u-u_{0})z}{f_{x}}\\ y=\frac{(v-v_{0})z}{f_{y}} \end{array}\right.$$

where A, B, C, D are the light plane coefficients; K is the camera intrinsic matrix; (u_0_, v_0_) are the principal point coordinates; fₓ and fᵧ are the focal lengths in pixel units; (u, v) are the image pixel coordinates; (x, y, z) are the 3D camera coordinates; and λ is a scale factor.

### 3.5. Point Cloud Preprocessing

To reduce noise and sparsity in the reconstructed point cloud while maintaining computational efficiency, three preprocessing steps were applied sequentially:

(1) Voxel down-sampling with a leaf size of 1.15 mm, to reduce point cloud density and accelerate subsequent processing.
(2) Statistical outlier removal based on 50 nearest neighbors and a standard deviation threshold of 1.0, to eliminate isolated noise points. These noise points mainly originate from weak reflections caused by the leaf veins under laser illumination.
(3) MLS up-sampling, which combines MLS smoothing and up-sampling into a single step (MLS search radius = 4.2 mm, up-sampling radius = 2.0 mm, step size = 1.5 mm). This step converts the sparse grid-like point cloud into a denser and more continuous surface representation of the cotyledon, which is critical for subsequent growth point localization.

**Figure 6:**
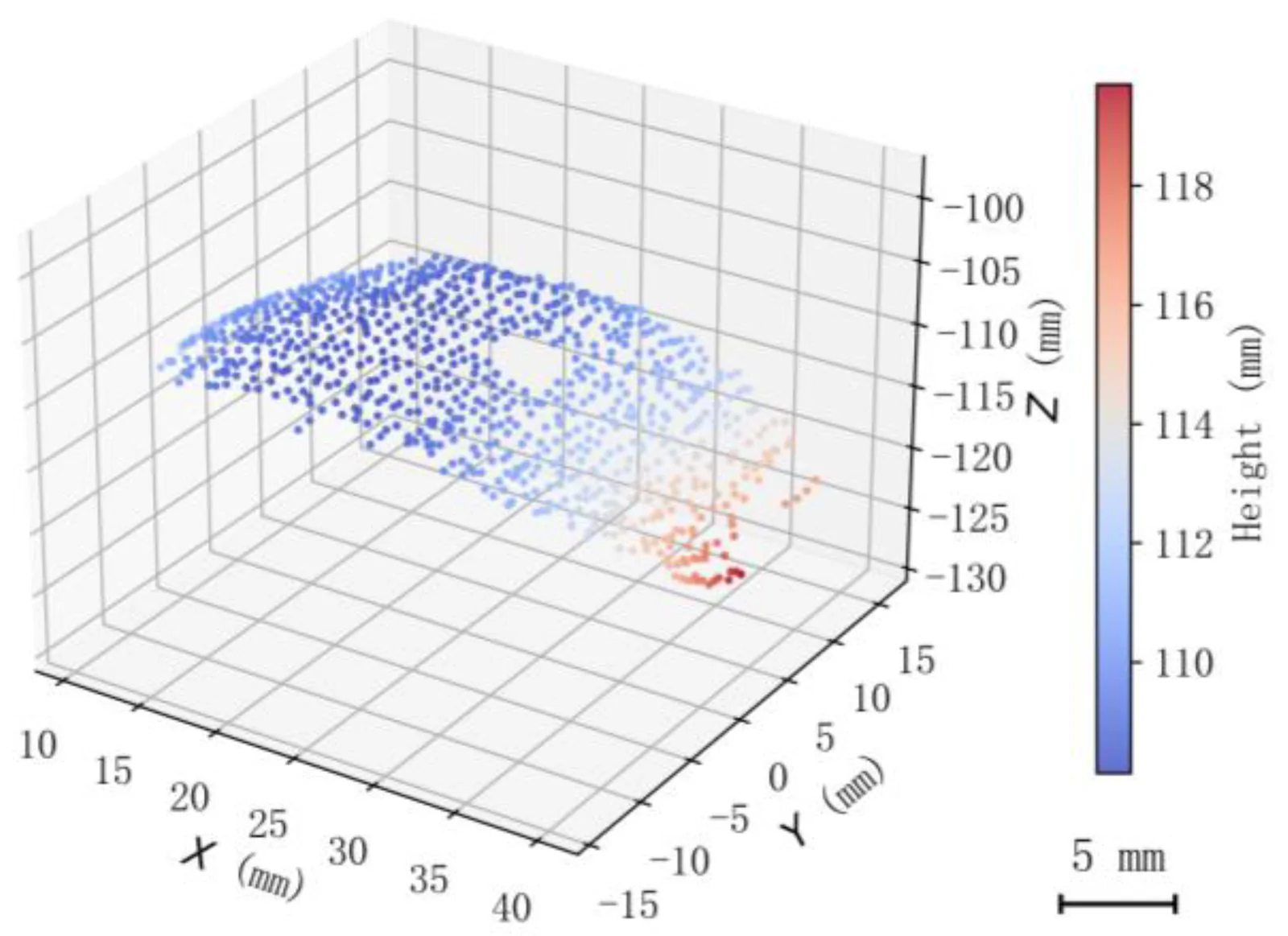
Preprocessed cotyledon point cloud.

The resulting preprocessed cotyledon point cloud is shown in Figure 6.

## 4. Growth Point and Growth Direction Recognition Based on Cotyledon Point Cloud

Due to the coding strategy limitations of the grid-structured light method, the current system can only reconstruct the point cloud of a single cotyledon (i.e., the upper cotyledon) and cannot simultaneously obtain the complete information of both cotyledons. Consequently, traditional growth point and growth direction recognition methods based on dual-ellipse fitting are not applicable to this system.

The fundamental reason lies in the projection and reconstruction mechanism of non-coded grid-structured light. Since the laser projector is positioned above the seedling, the lower cotyledon is completely occluded by the upper cotyledon in the top-down view. No laser lines are projected onto its surface, and thus no point cloud can be reconstructed for the lower cotyledon. Attempting to fuse the point clouds of the upper and lower cotyledons would introduce two major difficulties. First, the missing lower cotyledon data would have to be inferred from incomplete geometric cues, which is inherently ambiguous. Second, the fused point cloud would contain inconsistent surface normals and an ill-defined growth direction, making dual-ellipse fitting or any two-cotyledon fusion method unreliable. Therefore, the proposed triangle-approximation method is specifically designed for the single fan-shaped point cloud corresponding to the unoccluded cotyledon region that is illuminated by the grid laser pattern (typically the upper cotyledon). The fan shape naturally arises from the projection of the grid pattern onto the curved cotyledon surface, and its arc center consistently corresponds to the growth point, enabling robust single-leaf localization.

Observations showed that the point cloud obtained by taking the lowest point of the cotyledon root region as the sphere center and cutting with a suitable radius exhibited a fan-shaped (umbrella-like) distribution in the top-down projection. The approximate center of this fan-shaped region was close to the growth point. Based on this observation, a method that constructs a circumscribed triangle to approximate the fan-shaped point cloud and thereby locates the growth point is designed.

First, principal component analysis (PCA) was performed on the cotyledon point cloud. The direction of the first principal component was taken as the growth direction of the cotyledon (from the root to the tip).

Taking the point cloud center as the starting point, a central axis was constructed along the growth direction. A distance threshold of 2 mm was set, based on tests of d = 1, 2, and 4 mm on the same seedling dataset; d = 1 mm failed to cover the complete root region, while d = 4 mm included excessive background points, and d = 2 mm provided the best coverage of the root region. The points near the axis were extracted to form an axial point cloud strip. The average heights (Z values; the camera optical axis points downward along the positive Z direction, so a larger Z value indicates a lower height) at both ends of this axial strip were calculated. The end with the larger Z value was identified as the root region. Within the root region, the point with the maximum Z value, denoted as P_0_ (the lowest point, close to the growth point), was found. Using P_0_ as the sphere center, a radius of r = 50 mm was used to extract the points inside the sphere to form a fan-shaped local point cloud. This radius was determined based on tests on 10 sample seedlings. We evaluated r = 40, 50, and 60 mm. With r = 40 mm, the extracted fan-shaped point cloud was too small, leading to unstable results and a drop in detection accuracy. With r = 60 mm, the accuracy was comparable to that of r = 50 mm, but the computational time increased slightly. Considering both accuracy and efficiency, r = 50 mm was chosen as the final value, as it consistently extracted a complete fan-shaped point cloud in the root region sufficient for subsequent triangle fitting. This point cloud was then projected onto a 2D plane (top-down view) to facilitate subsequent geometric calculations.

**Figure 7:**
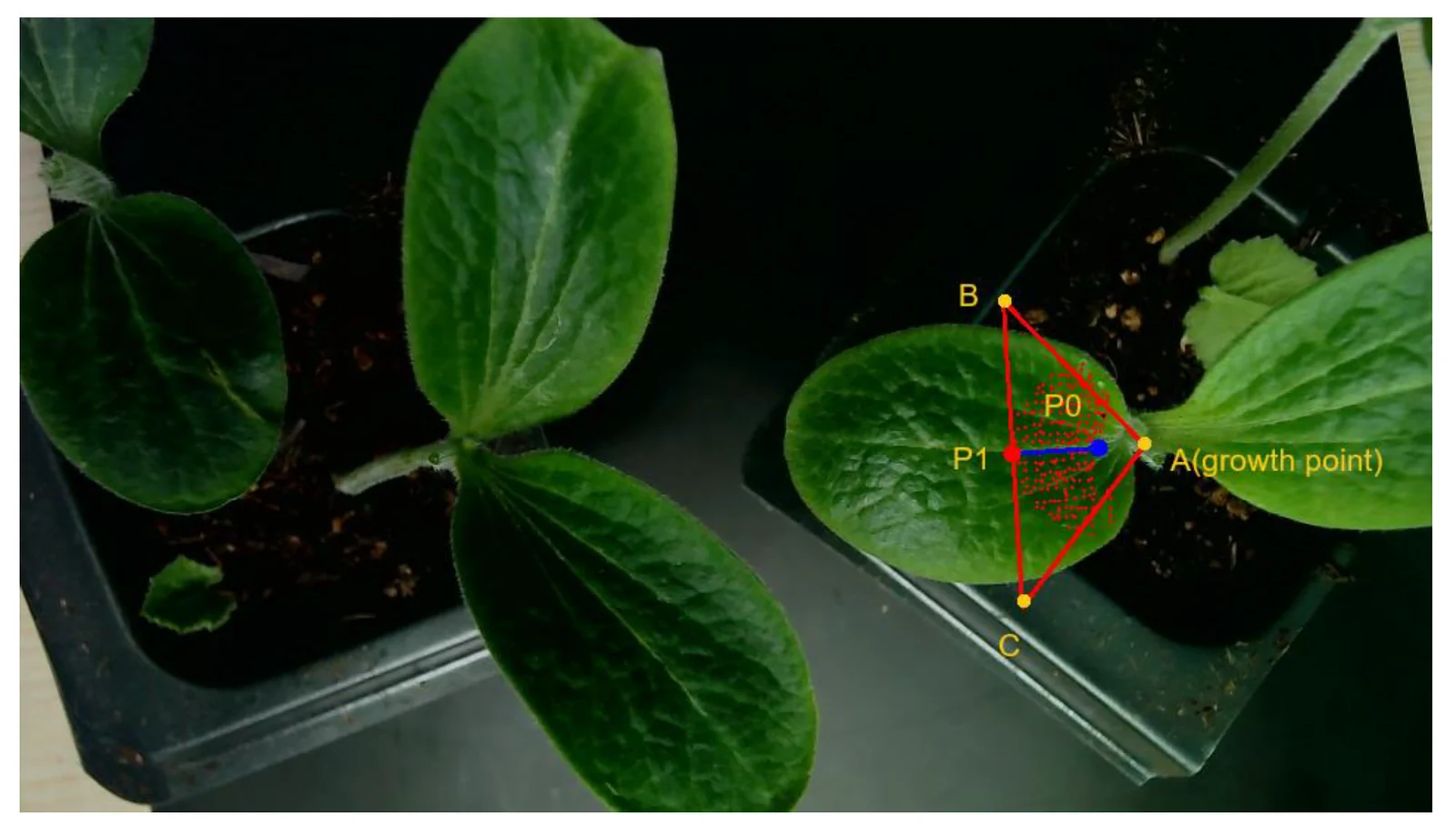
Triangle approximation for growth point estimation.

To locate the arc center of the fan-shaped point cloud (i.e., the growth point), a minimum triangle that encloses the fan-shaped point cloud is constructed, and the vertex closest to the arc center is taken as the growth point. The naming of key geometric elements and the construction of the triangle are illustrated in Figure 7. The construction procedure is as follows:

Step 1: Determine the fan boundary point $P_{1}$. In the fan-shaped point cloud, find the point with the largest projected distance from $P_{0}$ along the growth direction; this point is denoted as $P_{1}$.

Step 2: Construct the base edge BC. Draw a line through $P_{1}$ perpendicular to $P_{0}P_{1}$; the line segment BC on this line serves as the base of the triangle. The length of this segment should be greater than the width of the fan-shaped point cloud in the perpendicular direction. In this paper, ∣BC∣=1.2×W, where W is the maximum width of the fan-shaped point cloud in the direction perpendicular to the growth direction.

Step 3: Slide vertex A. Slide vertex A along the $P_{0}\to P_{1}$ direction with a fixed step size to construct triangles ABC. For each triangle, calculate the proportion of points from the fan-shaped point cloud that fall inside the triangle; this proportion is denoted as the enclosure ratio η.

Step 4: Determine the growth point. The localization error of the growth point was evaluated experimentally at different η thresholds (80%, 85%, 90%, 95%). The results show that the localization accuracy was optimal when η=90%. The vertex A satisfying this condition is taken as the growth point, corresponding to the arc center of the fan-shaped point cloud.

## 5. Experiments and Results

### 5.1. Light Plane Calibration Accuracy

The purpose of this experiment is to verify the influence of light plane calibration accuracy on point cloud reconstruction. The experimental platform consisted of the acquisition system described in Section 2.1, a high-precision *Z*-axis lift (accuracy 0.01 mm), and a black background board. The lift was moved stepwise with a 0.5 mm step to five different positions (from 5.0 mm down to 3.0 mm). At each position, a grid laser image was captured and a point cloud was reconstructed. For each plane, the mean Z-value, standard deviation of Z-values, and inter-plane errors were calculated. The results are listed in Table 2.

**Figure 8:**
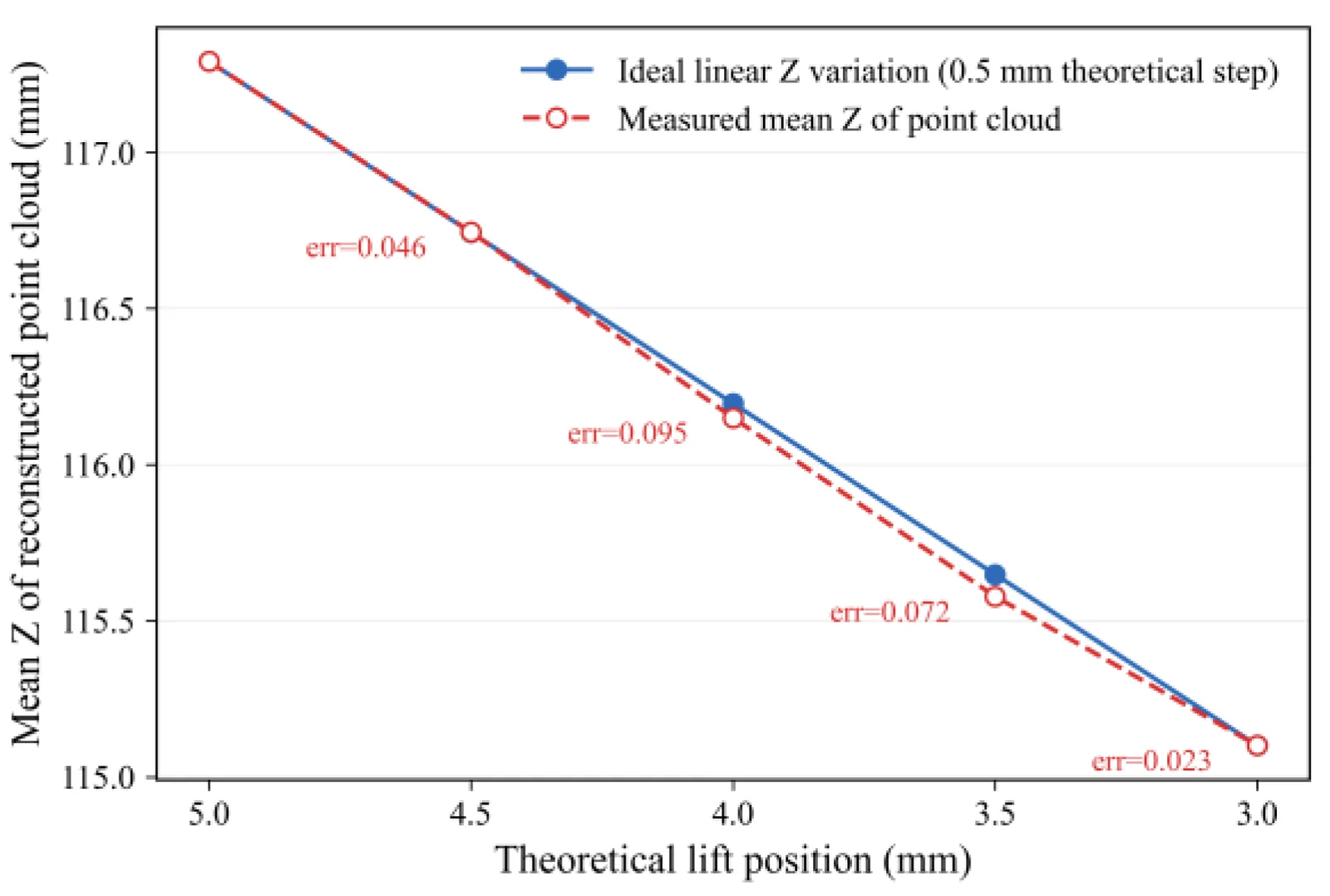
Comparison between theoretical lifting heights and measured Z-values of the point cloud.

The standard deviations of the Z-values of all planes ranged from 0.401 mm to 0.417 mm, indicating good flatness. The average height difference between adjacent planes was 0.548 mm, close to the theoretical step of 0.5 mm, and the average inter-plane error was 0.059 mm. The comparison between the theoretical lifting heights and the measured Z-values is visualized in Figure 8.

These results demonstrate that the light plane calibration accuracy meets the requirements for subsequent detection tasks.

### 5.2. Growth Point Detection for Single Non-Overlapping Seedlings

This experiment verifies the growth point localization accuracy of the proposed method on single plug seedlings. Fifty pumpkin rootstock seedlings at the cotyledon stage (approximately 7–10 days after germination) were selected. The cotyledon point cloud was reconstructed using the method described in Section 2, and the growth point coordinates were estimated using the method described in Section 3. The true growth point positions were manually marked on the original images and converted to world coordinates. The localization error was defined as the Euclidean distance between the estimated point and the true point.

**Figure 9:**
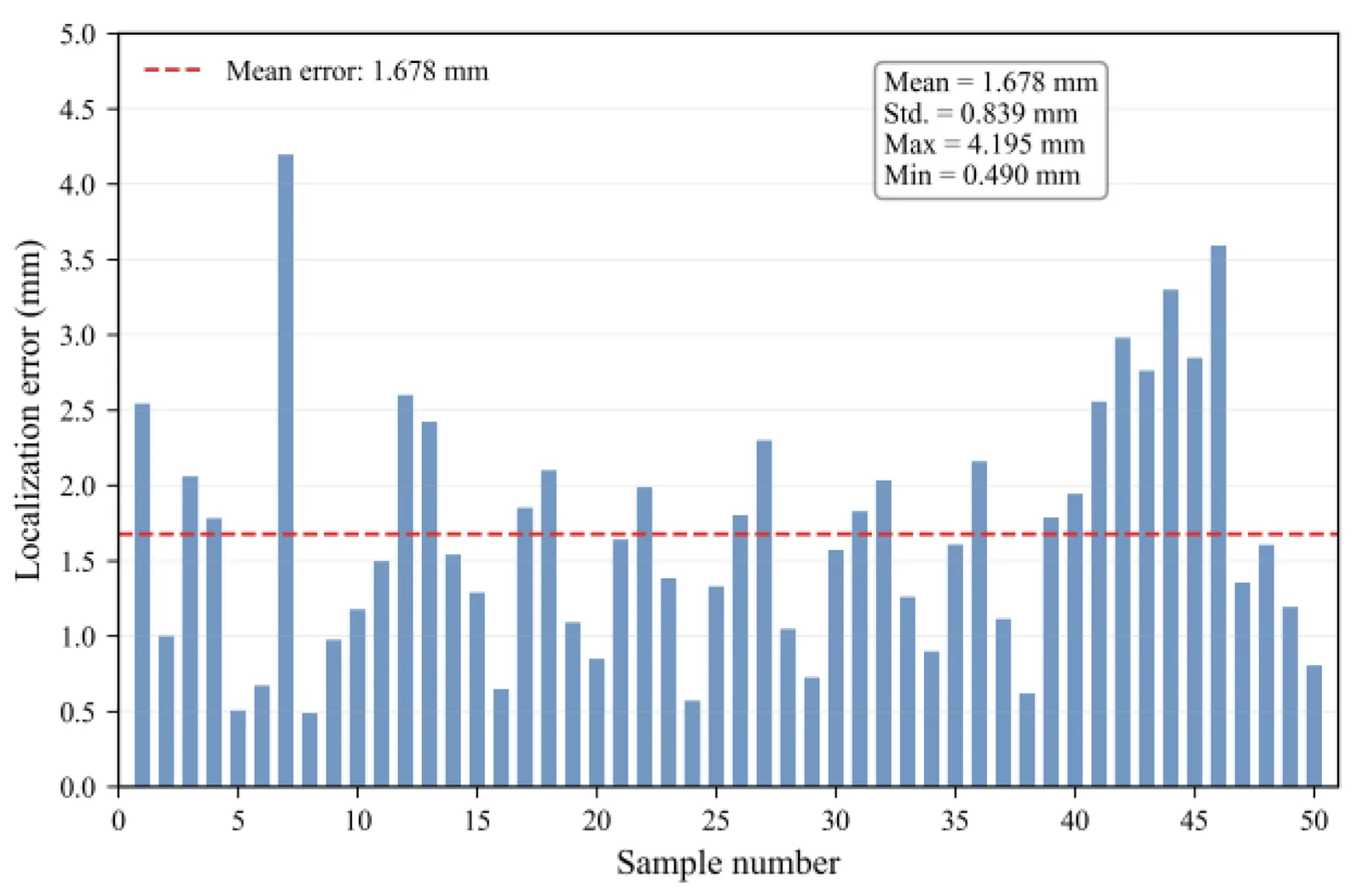
Distribution of growth point localization errors for 50 single seedlings.

The experimental results show that the overall average localization error was 1.68 mm, with a standard deviation of 0.91 mm. The maximum error was 4.19 mm, and the minimum error was 0.49 mm. The localization error of all samples was below 5 mm, corresponding to a success rate of 100%. Among them, 88% of the samples had an error within 2.5 mm. Figure 9 shows the distribution of localization errors for the 50 samples.

To evaluate the contribution of each of the three point cloud preprocessing steps described in Section 3.5 to the final localization accuracy, an ablation study was conducted on the same 50 single-seedling dataset. Three variants were tested, each omitting one of the three steps, and compared with the full three-step pipeline. The results are summarized in Table 3.

The ablation results show that each of the three point cloud preprocessing steps contributes to the overall performance. The MLS up-sampling step has the most significant impact on accuracy: without it, the localization error increases from 1.68 mm to 5.32 mm, confirming that converting the sparse grid-like point cloud into a dense continuous surface is essential for growth point localization. Voxel down-sampling has a relatively minor effect on accuracy (1.68 mm vs. 1.71 mm), but it substantially improves processing efficiency (reducing the time from 849 ms to 152 ms), which is important for real-time deployment. Statistical outlier removal provides moderate noise suppression, with a slight increase in error (1.92 mm) when omitted.

The average processing time per seedling was measured on a platform with an Intel Core i5-12400F processor and 16 GB RAM. The breakdown of the main processing stages is summarized in Table 4.

These results indicate that, in the single non-overlapping seedling scenario, the proposed method achieves a growth point localization accuracy that satisfies the requirements for robotic arm gripping.

### 5.3. Comparative Experiment for Overlapping Seedlings

To evaluate the robustness of the proposed method under occluded conditions, 150 plug seedlings with overlapping leaves were selected. Growth point detection was performed using both the proposed method (Section 2 and Section 3) and the traditional ellipse fitting method (reference [4]). The success rate was defined as the proportion of samples for which the output result had a localization error ≤ 5 mm compared to manual annotation.

**Figure 10:**
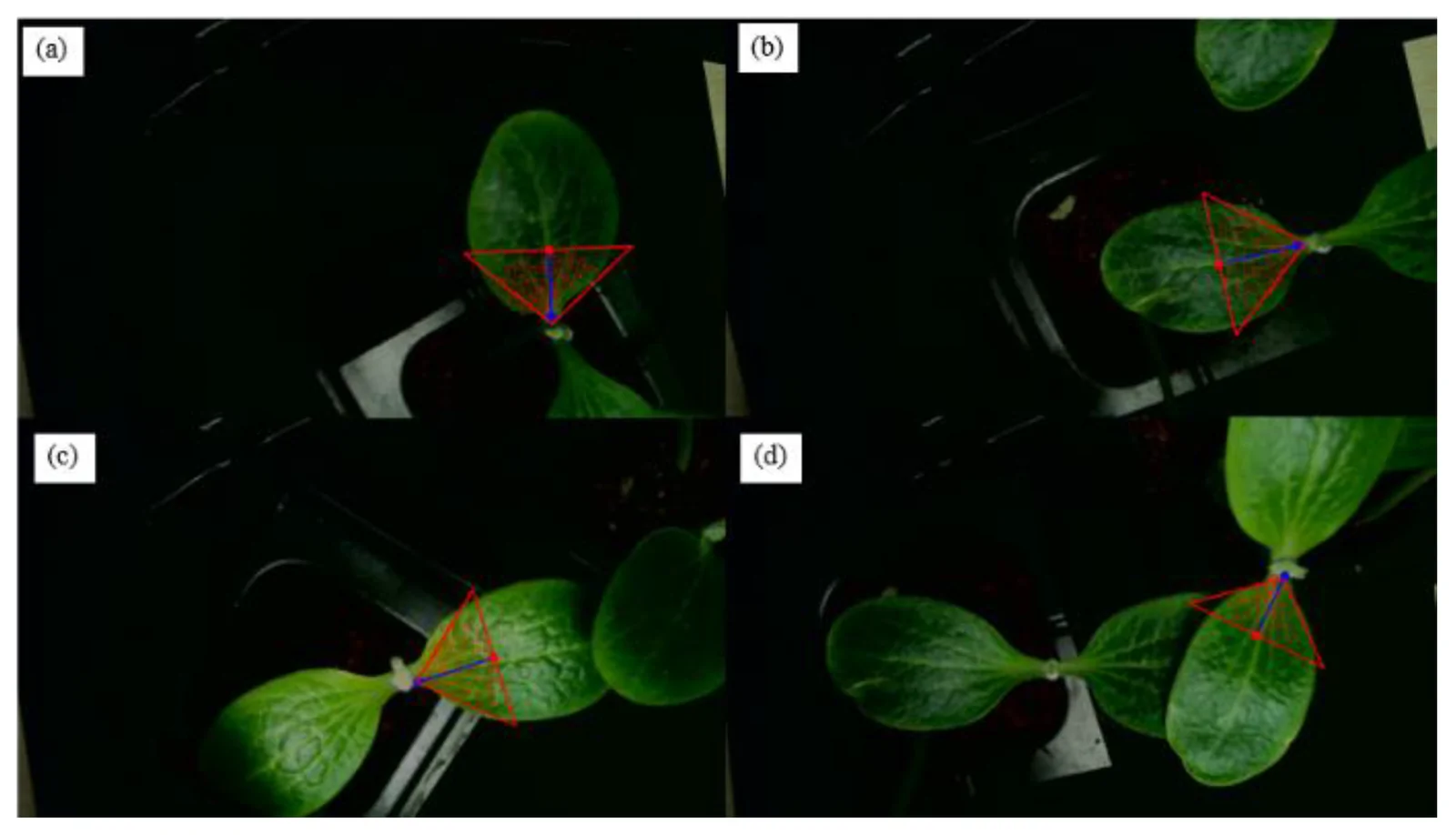
Examples of growth point detection results: (**a**) single seedling case 1; (**b**) single seedling case 2; (**c**) overlapping case 1; (**d**) overlapping case 2.

The experimental results show that the traditional ellipse fitting method was severely affected by contour interference under leaf overlap, successfully detecting only 102 out of 150 groups, corresponding to a success rate of 68%. In contrast, the proposed method, which reconstructs only the point cloud of the upper cotyledon using grid-structured light, effectively avoids interference from lower leaves and successfully detected 134 groups, achieving a success rate of 89%. Partial growth point detection results are shown in Figure 10.

**Figure 11:**
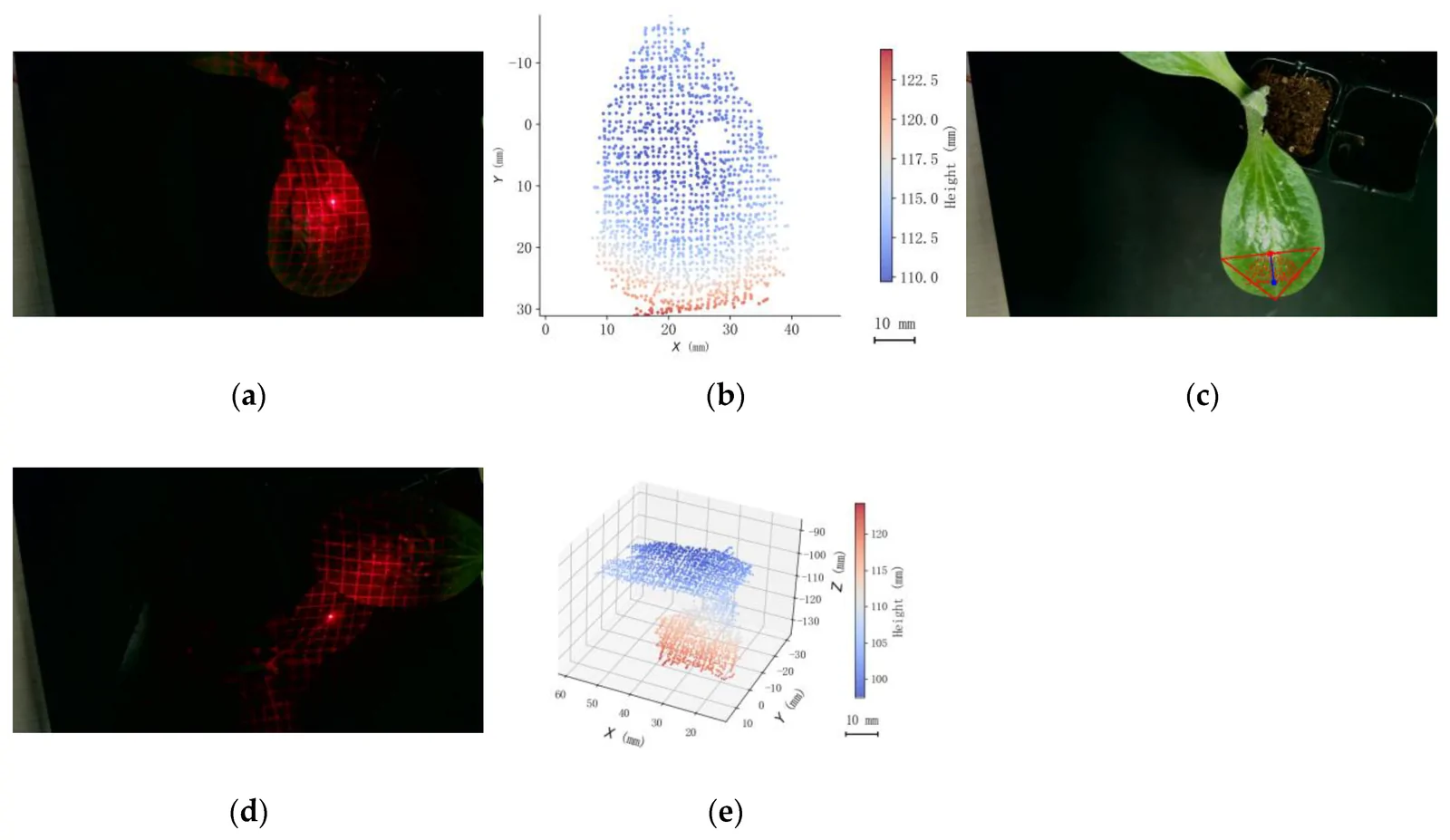
Typical failure cases of the proposed method: (**a**–**c**) failure caused by excessive leaf curvature, where (**a**) original image, (**b**) point cloud depth map, and (**c**) incorrectly localized growth point; (**d**–**e**) failure caused by merging of laser lines on overlapping cotyledons, where (**d**) laser image and (**e**) point cloud depth map.

The failure cases were mainly attributed to two factors. The first was excessive leaf curvature, where the point cloud height at the leaf tip became lower than that at the root region, making it impossible to correctly identify the root region based on height information and resulting in a “false growth point” on the opposite side. As shown in Figure 11a–c, this type of failure occurred in 6 out of 150 samples, indicating a relatively low but non-negligible occurrence. The second factor was the merging of laser lines on the surfaces of two overlapping cotyledons, which caused the extracted point cloud to contain mixed surface information from both cotyledons and prevented the PCA from determining a valid growth direction. Typical examples of this failure mode are illustrated in Figure 11d,e.

Further examination of the failed samples reveals that neither failure mode can be easily described by a single geometric threshold. The first mode (excessive leaf curvature) occurs when the leaf tip becomes lower than the root region in the top-down projection, so that the root region no longer appears as the local lowest point, making height-based root region identification impossible. The second mode (laser line merging) occurs when two cotyledons come into physical contact in 3D space, rather than merely overlapping in the top-down view. In such cases, the laser lines pass continuously across the contacting surfaces and are extracted as a single intact line, preventing the coding strategy from separating the point clouds of the two cotyledons. This leads to mixed point clouds and subsequent failure of PCA-based growth direction estimation. Due to the limited number of failed cases, this study does not provide quantitative geometric thresholds for these two failure modes. However, the qualitative observations reported here offer useful insights for future algorithmic improvements.

In summary, the proposed method shows clear advantages under occluded conditions. It should be noted, however, that the current method cannot detect completely occluded lower cotyledons. This is a limitation of the present approach. Future work could integrate multi-view imaging or deep learning methods to further improve performance.

## 6. Conclusions

This paper addresses the problem of growth point localization for plug seedlings and proposes a detection method based on grid-structured light. The main contributions and conclusions are summarized as follows:

(1) A grid-structured light image acquisition system was constructed, and a multi-depth-plane-based light plane calibration method was proposed. Experimental results show that the average inter-plane calibration error is 0.059 mm, which meets the accuracy requirements for 3D reconstruction.
(2) To handle the fracture and distortion of grid laser lines on cotyledon surfaces, a laser line coding method based on three-dimensional constraints of the light planes was developed. By matching the 3D consistency of intersection points, this method achieves robust coding of incomplete grid lines.
(3) Based on the fan-shaped structure of the cotyledon point cloud, a triangle-approximation method for growth point localization was proposed. Experiments on 50 non-overlapping single seedlings yielded an average localization error of 1.68 mm and a success rate of 100%.
(4) Comparative experiments on 150 overlapping seedlings show that the proposed method achieves a success rate of 89%, outperforming the traditional ellipse fitting method (68%), thereby demonstrating its robustness under occluded conditions.

Future work will focus on the detection of completely occluded lower cotyledons. Several concrete directions will be explored: first, multi-angle grid-structured light fusion by placing multiple laser projectors around the seedling to capture occluded surfaces; second, semantic segmentation using deep learning to separate mixed point clouds of overlapping cotyledons; and third, lightweight neural networks for real-time laser line coding to reduce the processing time for online deployment.

## Figures and Tables

**Table 1:** Camera intrinsic parameters and distortion coefficients ($k_{1}$, $k_{2}$: radial; $p_{1}$, $p_{2}$: tangential).

Parameter	Value	Parameter	Value
$f_{x}$	731.3271	$f_{y}$	731.0710
$c_{x}$	475.5218	$c_{y}$	291.3238
$k_{1}$	0.0824	$k_{2}$	−0.0671
$p_{1}$	0.0009	$p_{2}$	−0.0002

**Table 2:** Experimental results of light plane calibration accuracy.

Test Plane No.	Theoretical Position of Lift (mm)	Mean Z of Point Cloud (mm)	Standard Deviation of Z (mm)	Inter-Plane Error (mm)
1	5.00	117.291	0.401	-
2	4.50	116.745	0.414	0.046
3	4.00	116.150	0.417	0.095
4	3.50	115.578	0.408	0.072
5	3.00	115.101	0.405	0.023

**Table 3:** Ablation study of point cloud preprocessing steps.

Configuration	Mean Localization Error (mm)	Mean Point Cloud Processing Time (ms)
Full three-step pipeline	1.68	152
w/o voxel down-sampling	1.71	849
w/o statistical outlier removal	1.92	151
w/o MLS up-sampling	5.32	10

**Table 4:** Runtime breakdown of the proposed method per seedling.

Stage	Time (ms)
Laser line extraction	131
Laser line coding	50
Point cloud generation	23
Point cloud preprocessing	152
Growth point detection	12
Total	368

## Data Availability

The data presented in this study are available on request from the corresponding author. The data are not publicly available due to privacy restrictions.
